# Non-Equilibrium Crystallization of Monotectic Zn-25%Bi Alloy under 600 *g*

**DOI:** 10.3390/ma14154341

**Published:** 2021-08-03

**Authors:** Grzegorz Boczkal, Pawel Palka, Piotr Kokosz, Sonia Boczkal, Grazyna Mrowka-Nowotnik

**Affiliations:** 1 Faculty of Non-Ferrous Metals, AGH University of Science and Technology, 30-059 Krakow, Poland; pawel.palka@agh.edu.pl (P.P.); piotrek.kokosz@gmail.com (P.K.); 2 Łukasiewicz Research Network—Institute of Non-Ferrous Metals, Division in Skawina, ul. Pilsudskiego 19, 32-050 Skawina, Poland; sboczkal@imn.skawina.pl; 3 Department of Material Science, Rzeszów University of Technology, Al. Powstańców Warszawy 12, 35-959 Rzeszów, Poland; mrowka@prz.edu.pl

**Keywords:** supergravity crystallization, gravitational segregation, texture, hexagonal alloys, monotectic transformation

## Abstract

This study investigated the influence of supergravity on the segregation of components in the Zn–Bi monotectic system and consequently, the creation of an interface of the separation zone of both phases. The observation showed that near the separation boundary, in a very narrow area of the order of several hundred microns, all types of structures characteristic for the concentration range from 0 to 100% bismuth occurred. An additional effect of crystallization in high gravity is a high degree of structural order and an almost perfectly flat separation boundary. This is the case for both the zinc-rich zone and the bismuth-rich zone. Texture analysis revealed the existence of two privileged orientations in the zinc zone. Gravitational segregation also resulted in a strong rearrangement of the heavier bismuth to the outer end of the sample, leaving only very fine precipitates in the zinc region. For comparison, the results obtained for the crystallization under normal gravity are given. The effect of high orderliness of the structure was then absent. Despite segregation, a significant part of bismuth remained in the form of precipitates in the zinc matrix, and the separation border was shaped like a lens. The described method can be used for the production of massive bimaterials with a directed orientation of both components and a flat interface between them, such as thermo-generator elements or bimetallic electric cell parts, where the parameters (thickness) of the junction can be precisely defined at the manufacturing stage.

## 1. Introduction

Metal crystallization is a well-known, natural process that has been modelled under equilibrium conditions. However, moving away from equilibrium conditions leads to interesting effects, such as inhomogeneous chemical composition, the formation of metastable phases and the displacement of phase transition points. The research carried out by other authors so far includes the study of the effect of the crystallization rate in microgravity conditions on the morphology of the precipitates [1] as well as microstructure refinement in the magnetic field [2].

Hence, the idea to investigate the alloys previously tested under these conditions in supergravitation conditions has appeared. The presented experiment is of a cognitive nature. The processes taking place during the gravitationally oriented crystallization have a universal character and can also be applied to other alloys. The obtained knowledge will allow a broader understanding of the mechanisms responsible for a forming of the alloy microstructure in non-equilibrium conditions.

The direct transfer of the results of the experiment to the real technology requires further work, although a similar method is used in the centrifugal casting process. It should also be mentioned that the conditions described in the experiment, which are extreme on Earth habitats, might be normal on other places in space. The current technological expansion and solar system exploration require new information about material responses under various conditions, from microgravity in Earth orbit to overload effects, which are given elements of spacecraft.

The overload used in the experiment, resulting from rotational motion, is the equivalent of the gravitational force of gravity multiplied by 600×. One of the research studies concerning the influence of centrifugal force on the crystallized structure of alloy was carried out by Lőffler et al. and its results were published in 2002–2004 [3,4]. In the experiment, two magnesium (Mg)-based alloys and one aluminum (Al)-based alloy were melted and simultaneously rotated. The inertial acceleration was 60,000 *g*, where *g* is gravitational acceleration. As a result of these unique conditions, the researchers obtained samples where the gravitational segregation of phases could be observed. The primary phases sedimented at the end of the samples, while binary and ternary eutectics were solidified in the central regions of the samples. The authors of the experiment hoped that their research would help to discover a new type of bulk metallic glasses. Centrifugal force can also be used to purify metal. Ying et al. [5] carried out research on a lead (Pb) alloy with 3% the weight of copper (Cu). The researchers managed to get 1000 *g* which caused significant segregation of Cu in the upper part of the tested sample. The volume fraction of Cu changed from 15.57% in the top of the sample to 0% in the bottom. Another attempt to purify metal using supergravity was taken up by Lixin Zhao et al [6]. In this research, industrial Al was purified by gravitational segregation. The scientists obtained 400 *g* and the concentration of iron (Fe) changed from 3.5 to 1.5 weight percent between the bottom and top of a sample, which was 1.5 cm long. The same group of scientists suggested that supergravity could be used for grain refining [7]. In the experiment, scientists crystallized industrial Al with different gravity coefficients (ratio of super-gravitational acceleration to normal gravitational acceleration). It was observed that the average grain size decreased exponentially and after reaching 250 *g*, the average grain size was constant.

Phase separation during the sedimentation process in Cu–Tin (Sn) alloy under supergravity was investigated in 2019 by Wierzba et al. [8]. Centrifugal casting is used in industry; the main application is the improvement of the filling of molds [9,10]. It is also used in the production of metallic glasses [11]. The described method engages changes in the parameters of nucleation kinetics caused by high overloads. By crystallizing a material under high overload, one can potentially control its structure. Partial amorphization, mentioned in the paper [11], is a consequence of a decrease in thickness of the formed lamellar eutectic phases. At sufficiently small thickness of eutectic lamellas, certain chemical composition and appropriate cooling rate, the conditions for partial amorphizations in the microstructure can be fullfiled. On the other hand, after changing the parameters, it is possible to obtain highly ordered structures of quasi-crystalline character.

There are reports of the use of this method to produce highly ordered structures, not only based on metals [12]. As a consequence of the above considerations, the Zinc (Zn)–Bismuth (Bi) alloy was selected for research due to the simple equilibrium system and the low solidus temperature. It has also not been previously investigated in non-equilibrium crystallization conditions, especially in supergravity crystallization conditions.

### 1.1. Investigations of the Zn–Bi Alloys

This article focuses on the effect of centrifugal force on the crystallization process of Zn-25%Bi alloy. Low mutual solubility of both metals should generate a microstructure with regular precipitates, convenient for analyses. The proportions of the components in the alloy were selected on the assumption that strong gravitational segregation would lead to the generation of a wide spectrum of microstructures in the material, characteristic for various compositions of the alloy.

Previous results obtained by the authors for other zinc alloys showed a tendency to grow a preferred orientation along $\langle 11\bar{2}0\rangle$ direction during zone crystallization [13,14]. It was a concept that similar effect could occur in the supergravity solidification of hexagonal alloys. 

Different aspects of Zn–Bi alloys were investigated before and described in literature.

One of the research concepts was crystallization under microgravity. In zinc-rich content alloys, Bi precipitates have had a drop shape and their sizes were larger than from diffusion-controlled growth. A concentration of more than 24% Bi in samples congregate in large (Bi) areas in the center of the sample and generate a (Bi) halo-layer around the sample [15].

Different aspects of Zn–Bi alloy research were presented in work [16]. The authors applied the squeeze casting method for the production of the alloy chips. Crystallization proceeds under pressure, 120 MPa, until complete solidification. The obtained material showed increases in density, tensile strength and hardness. Their electrical resistivity decreased [16].

Moreover, bismuth as a metal is a potential substitute for lead in low-melting solders and was described in a phase control method in the Zn–Bi alloy by tungsten nanoparticles [17]. The effect was refined for the microstructure and to avoid sedimentation.

Zinc–bismuth alloys are also interesting for their bactericidal applications [18]. This is allowed for improvement of typical anticorrosion zinc covers with bactericidal and antifungal effect. 

These facts motivated the authors to expand their knowledge of this interesting alloy to include the effect of supergravity on crystallization. This aspect has not been previously studied for monotectic systems.

### 1.2. Zn–Bi System Characteristic

**Figure 1:**
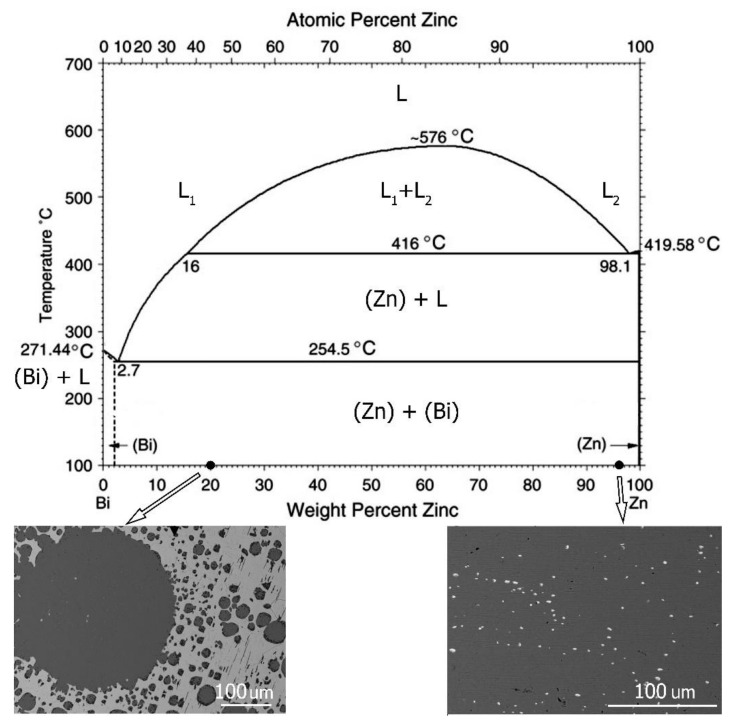
Bi–Zn phase diagram [19,20,21] and microstructures observed at different chemical compositions of investigated alloys.

The Zn–Bi phase system is shown in Figure 1. A characteristic feature of the system is the monotectic presence in the tested range of composition. Figure 1 shows the microstructure of various Zn–Bi alloys obtained under the conditions of equilibrium crystallization.

The alloy components differ in density, i.e., ρBi = 9.79 g/cm^3^, while ρZn = 7.14 g/cm^3^. This promotes the occurrence of the effect of gravitational segregation. A significant difference in the melting point of the components was reflected in the surface tension of the liquid: at 426 °C (419 °C is the melting temperature of zinc), its values are γZn = 810 mJ/m^2^ for Zn [22] and γBi = 360 mJ/m^2^ for Bi [23].

Microstructures obtained with different zinc contents are shown in Figure 1. At 20 wt% of Zn, spherical zinc phase precipitates were noted to occur in a (Bi) matrix. A characteristic feature was the large scatter of the values of the diameter of individual precipitates. Primary precipitates with diameters in the order of 200–300 microns and small precipitates with dimensions of several microns were directly adjacent to each other. It was the result of crystallization from the monotectic phase with a large difference in the surface tension of both liquids. Zinc was crystallized first and had a high surface tension, allowing for the formation of large spherical precipitates during primary crystallization. Additionally, minor secondary precipitates of Zn-rich needle-shaped phases were visible.

The second type of structure presented on the right side of Figure 1 contains 4 wt% Bi. The observed microstructure consisted of (Zn) matrix and small, spherical bismuth particles of similar size. The monotectic transformation occurring in the Zn–Bi system favors the spherical shape of the precipitates regardless of their chemical composition [10,24].

## 2. Materials and Methods

**Figure 2:**
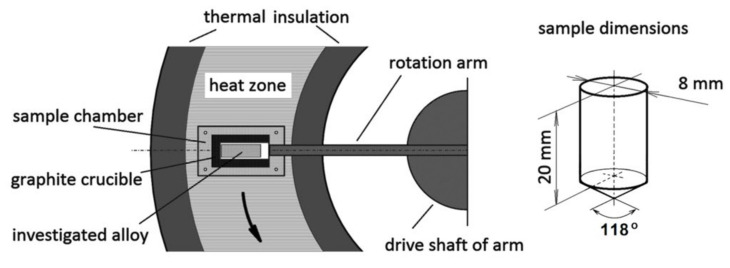
Scheme of the ring furnace and sample geometry. One experiment needs two identical samples in two chambers on both tips of arm.

Tests were carried out on a test stand designed and constructed for the purpose of this study. A furnace was made in a ring configuration, as is shown in Figure 2.

The furnace with heating elements is stationary, only the symmetric arm with two chambers on both sides rotates. The arm system is balanced and has bearings on both sides. The obtained parameters, such as supergravity and temperature were limited mainly by the strength of the material used for the arm and crucible. At a temperature of 500 °C and a machine operation cycle of about 3 h, the material of the arm is exposed to creep processes and therefore, the arm has been made of high alloy steel. The crucible material also imposed some constraints. The tests were carried out using graphite chambers, but it became necessary to use disposable elements made in the form of a monolith with a cylindrical chamber of 8 mm diameter and a length of up to 20 mm.

The motor used in the device had a nominal rotational speed of 1350 RPM and therefore, the samples were rotating with frequency of 1350/60 = 22.5 Hz. The correlation between frequency and angular velocity is given by the formula:

(1) ω=2πf

where: *f*—frequency s^−1^.

It can be calculated that the angular velocity of the sample was: $\omega =141.31\frac{rad}{s}$.

The radius of the circle in which the samples were moving was 300 mm. The radius was measured up to a central point of the sample, at a half-length. Using a simple formula for angular acceleration, it was possible to calculate it as equal to 5995 m/s^2^. The measure of the load exerted on the sample will be expressed in the angular acceleration divided by gravitational acceleration of 9.81 m/s^2^. The normal acceleration was 611.15 *g*. It was assumed that the arms had an angular velocity equal to the nominal speed of the motor used and the normal acceleration can be estimated at 600 *g* for the whole length of the sample. Due to the finite length of the sample, a slight overload gradient will be present along the length of the sample, hence the efforts to obtain the maximum long arm to reduce this gradient.

To prepare samples, measured amounts of the alloy were cast into the crucibles, followed by the balancing of the system. In the first step of the experiment, the electric motor was turned on and the arm with samples started to rotate. When the rotation speed (overload) was stabilized, the furnace was turned on. The furnace was heated up to 500 °C, which took 30 min. The samples were held at 500 °C for 30 min to make sure that they melted down completely, then the furnace was turned off and the samples cooled down with the furnace to 200 °C. Cooling the furnace to 200 °C took another 30 min and after that time, the rotation of the samples was stopped and they were taken out of the device and further cooled in the air.

The samples thus obtained were cut lengthwise. The cutting was done using a Struers Secotom-10 circular saw with a corundum disc. Cutting parameters were: disc rotation—2000 rpm, movement—0.05 mm/s, with cooling by lubricant mixture. The sample surface was then polished with diamond pastes and finally, with 0.04 microns graded alumina suspension.

Structural examinations were carried out using a Hitachi S-3400N scanning electron microscope (SEM, Hitachi High-Technologies Corporation, Tokyo, Japan) with an EDS Thermo Noran System Six chemical composition analyzer in microregions and an Inspect F50 with EBSD TSL detector (Edax Ametek, Tokyo, Japan). The EDS line scan step resolution was 68 micrometers and the measurement was performed at the accelerating voltage 20 kV with the acquisition time of 60 s per each measurement point (50 measurement points in line). Another SEM (Hitachi SU-70) equipped with wavelength dispersive spectrometer (WDS) and Ultra Dry by Thermo Noran was used to analyze fine Bi precipitates.

Hardness measurements were carried out by the Vickers method with a load of 0.19807 N, using a Innovatest Nova 240 microhardness tester (Innovatest, Maastricht, The Netherlands).

In total, 15 indentations were made in the Bi-rich zone and 30 indentations in the Zn-rich zone.

The phase composition studies were carried out using the Bruker D8 Advanced/Discover device, equipped with a copper tube. For improving the monochromatic radiation of wavelength λCuKα1 = 1.5406 Å, the Ni filter was used. The analysis of the phase composition of the tested materials was carried out using the PDF-2 crystallographic basis. The measurement was made with a scan step size of 0.02°. The following cards were used to analyze the phase composition: Zn: 00-004-0831 and Bi: 00-044-1246. 

Additionally, the same diffractometer was used to determine of pole figures from the planes (0002) and $\left(10\bar{1}0\right)$ for zinc and $\left(01\bar{1}2\right)$, $\left(20\bar{2}2\right)$ for bismuth. The measurement was performed by averaging the results of a scan of the longitudinal section of the sample from a length of 12 mm, thereby obtaining mean value of macro texture. Orientation distribution function (ODF) was determined using M-TEX software in the Matlab environment.

## 3. Results

### 3.1. Microstructures Analysis

**Figure 3:**
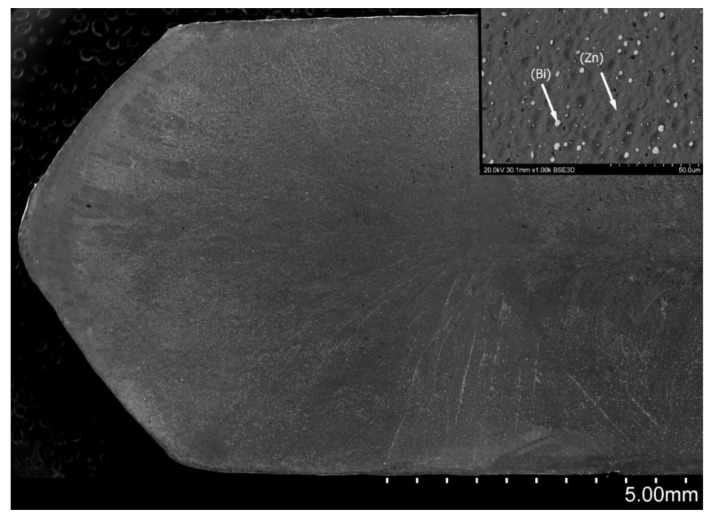
Microstructure of the Zn-25 wt% Bi alloy on longitudinal section after casting into the cold mold, with magnification of the representative microstructure containing (Bi) particles.

Figure 3 presents the Zn-25Bi alloy cast to mold at room temperature and crystallized under 1 *g* normal gravity.

Casting molds were made from cold graphite and were the same as during the supergravity test. Cooling processes were done in air. The cross-sectional microstructure was typical of a volume crystallizing casting. Crystallization in accordance with the directions of heat dissipation were visible. Bismuth was evenly distributed in a sample volume. Gravity segregation was not observed. 

**Figure 4:**
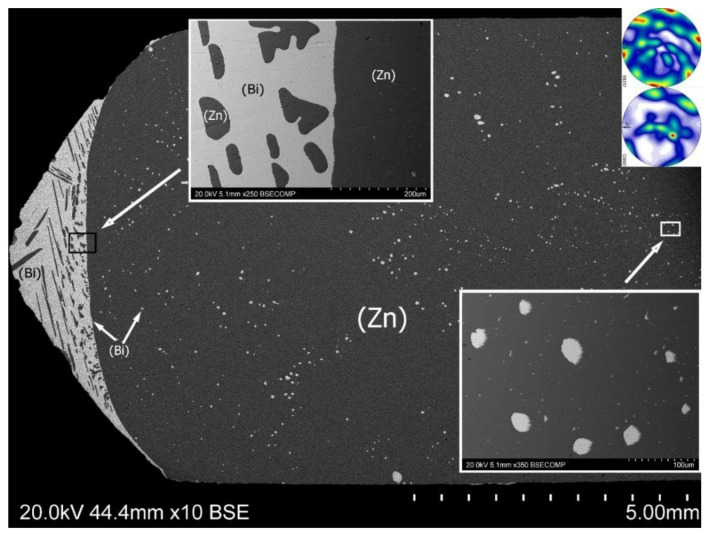
Microstructure of the Zn-25 wt% Bi alloy on longitudinal section, cooled with the furnace at normal gravity 1 *g*. Microstructures in two characteristic places are shown in higher magnification. Macro-textures from (Zn) phase are included in the right upper corner.

Figure 4 presents the alloy casted and crystallized under 1 *g* normal gravity, then cooled with the furnace. Long time exposition in the liquid state caused natural gravity segregation of components. A bismuth-rich and zinc-rich areas are visible in the microstructure. The separation line between them is a curve that represents a shape of a crystallization front.

The radius of the boundary can be interpreted as a characteristic parameter in relation to the cooling rate and temperature field.

Two observed zones represent individual microstructures. In the zinc-rich area, many spheroidal particles of almost pure bismuth can be observed. These are the products of the monotectic transformation taking place at the temperature of 416 °C under the conditions of thermodynamic equilibrium. Their different sizes and inhomogeneous distribution are a consequence of a coagulation effect during slow crystallization [24]. The larger surface tension of zinc, then bismuth at the same temperature and the almost lack of mutual solubility of both elements generated the observed shapes of Bi particles. 

The opposite effect took place for zinc precipitates into the (Bi) matrix. Zinc has higher melting point than bismuth, thus the embryo of (Zn) solid phase can grow as a primary precipitate. The growth orientation was limited by heat transfer directions. Moreover, the considerably larger size of the zinc precipitates was caused by a relatively long time of diffusion.

At the border of both zones, from the side of the zone rich in zinc, one can observe a strip devoid of bismuth precipitates. This effect is analogous to the halo effect observed around weakly coherent precipitates of slowly crystallized alloys. The same zone also exists for an alloy crystallized in supergravity conditions.

At the interface, from the bismuth-rich side, there are few irregular zinc precipitates with oval shapes.

**Figure 5:**
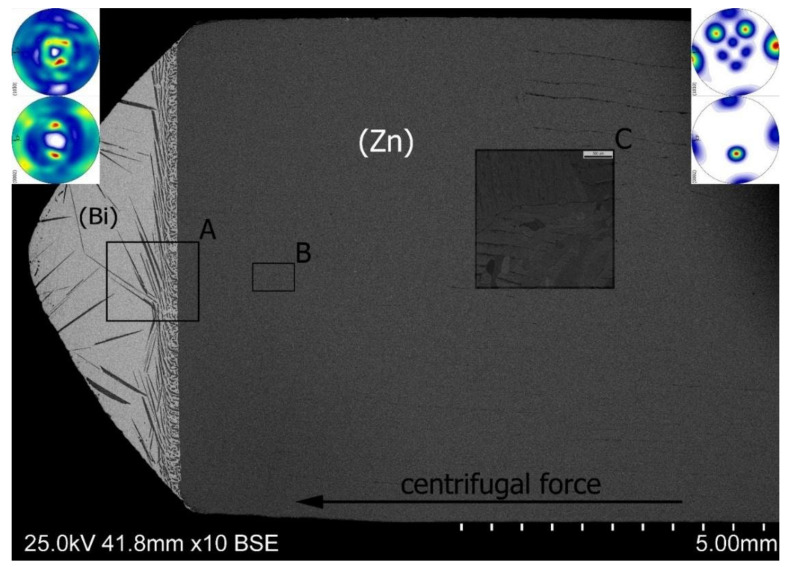
Microstructure of the Zn-25 wt% Bi alloy on longitudinal section along centrifugal force direction (600 *g*). Marked areas (A, B, C) in the figure are presented in a bigger magnification in the next figures. Macro-textures from: (Zn) phase are included in the right upper corner and (Bi) phase are included in the left upper corner.

Figure 5 shows a longitudinal section of the Zn-25 wt% Bi alloy sample crystallized under 600 *g* supergravity. Strong segregation has occurred and as a result, most of the bismuth was located in the left part of the sample (the bottom edge of the drawing is parallel to the centrifugal force direction). The segregation boundary between (Zn) and (Bi) zones is flat. This is a specific area with different chemical composition, changed along 1 mm distance. Both sides of this line concern alloy compositions from the opposite sides of the phase diagram. Non-equilibrium crystallization generates into this zone all possible morphologies for the Zn–Bi system.

**Figure 6:**
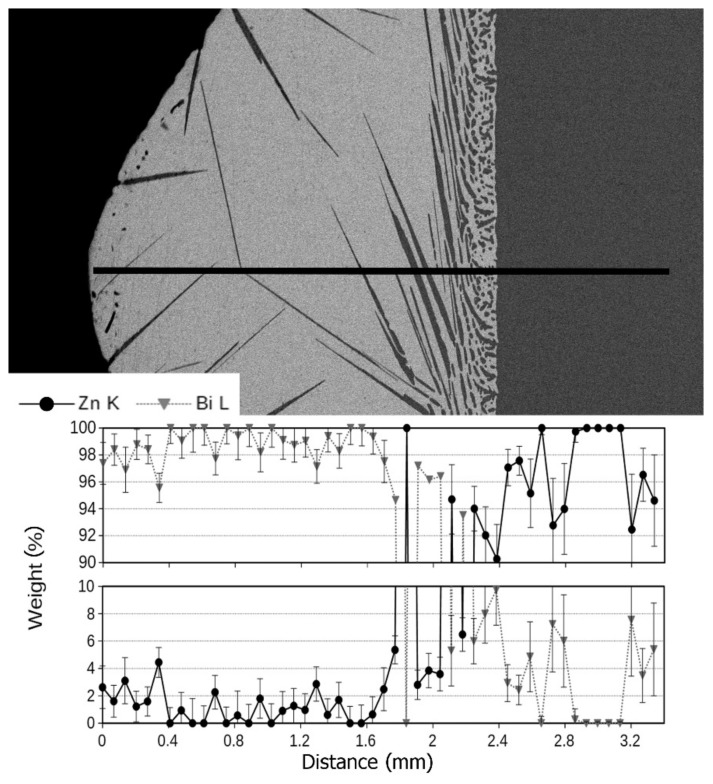
Linear EDS analysis of Bi and Zn content at the tip of the sample. Supergravity crystallization introduced a strong segregation effect.

To examine the mutual distribution of Zn and Bi, an EDS linear analysis of the chemical composition of the obtained structure was carried out along the centrifugal force direction as shown in Figure 6. 

The microhardness of regions rich in zinc and bismuth was measured. In the bismuth-rich zone, the average microhardness HV0.01 was equal to 17.6 with standard deviation of 4.9, while in the zinc-rich zone, the microhardness was 58.5 with standard deviation of 5.1.

The analysis of the macrostructure showed the existence of a few characteristic zones, marked A, B, C in Figure 5.

Moving from the left tip of the sample in Figure 5, one can see large needle-shaped precipitates formed as a result of the eutectic reaction; their eutectic point is located at 2.7 wt% of Zn and at temperature of 254.5 °C (Figure 1). The nucleation sites of the needles of a hypoeutectic primary phase located on the outer edges of the sample are very interesting. Their presence indicates heterogeneous nucleation on the walls of the crucible, while large dimensions of the particles suggest their growth from the liquid as a primary phase. Moving right towards the interface, an increase in the amount of zinc is visible (Figure 6).

**Figure 7:**
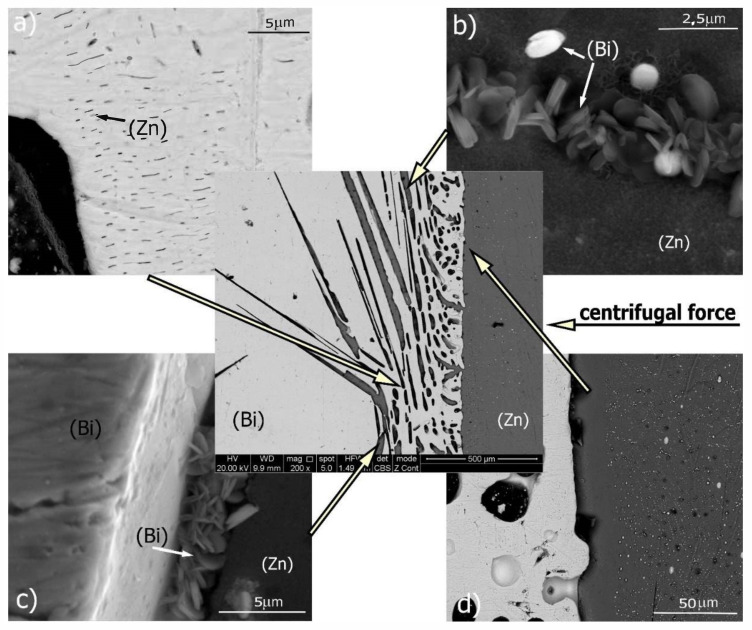
Interface between Bi-rich zone and Zn-rich zone (A-area in Figure 5). A few characteristic features can be observed: (**a**) very narrow zinc needles in Bi-rich zone, ordered parallel to G-force vector; (**b**,**c**) tiny lamellas of bismuth eutectic precipitates; and (**d**) a bismuth-free area on the Bi/Zn boundary.

Close to the sedimentation area, smaller and more densely distributed precipitates appeared, characteristic of structures with a chemical composition lying near the eutectic point (Figure 7, near separation zone). At the same time, small needle-shaped precipitates fill the spaces between larger crystallites. This is shown in Figure 7a. This type of structure can be observed in a relatively narrow range at the interface. The reason is to be searched in the specific character of the Zn–Bi system. In this system, the solidus line runs practically horizontally and only from the side of high bismuth contents does its temperature exceed 254.5 °C. This allows the liquid phase to maintain its presence up to this temperature, regardless of the chemical composition which, in turn, promotes the diffusion process and maintains the crystallization kinetics at a constant level throughout the entire length of the sample.

At the bismuth/zinc interface, small zinc-rich plate-shaped precipitates were observed (Figure 7b,c). The EDS analysis of chemical composition showed a zinc content of more than 70 wt%, but the small size of the particles and their geometry did not allow for more accurate determination. This indicates that a second reaction occurred at the monotectic point of 98.1 wt% Zn/416 °C. Columnar Zn crystals separated by higher Bi concentration regions could be imprints of the monotectic reaction. 

The chemical composition of the characteristic points in the Zn–Bi phase diagram was given on the basis of data from the equilibrium crystallizing system. In fact, the position of this point can be significantly shifted by the force of gravity. Moreover, along G-force direction, the density of alloy changed. It is a gravity segregation effect. The density change along the sample length occurred in a non-linear way, with a narrow zone on the border of the zinc and bismuth-rich areas, where the change has gradient character. Literature reports show that significant shifts of transition points on systems crystallizing under conditions of high pressure, for example, are possible [25,26,27,28].

The next detected area is shown in Figure 7d. This zone contains pure zinc and is practically devoid of bismuth. The width of the zone ranges from 10 to 20 microns. Its presence is due to the precipitation of primary phases from the solution taking place at the interface. The result is an effect similar to the “halo” phenomenon observed around the precipitates of the primary phases. It involves total removal of one of the alloy components from the area surrounding the precipitate [29].

**Figure 8:**
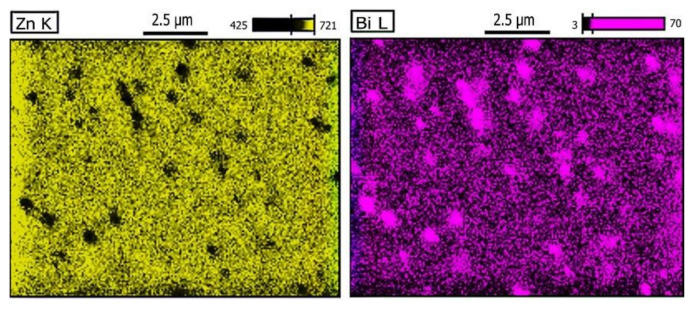
Wavelength dispersion spectroscopy (WDS) analysis in the Zn-rich area. In the zinc matrix, many dispersed (Bi) particles can be observed.

Moving to the right along the sample axis reveals the presence of a large amount of fine spheroidal precipitates of pure bismuth (zone B marked in Figure 5 and the left side in Figure 7d). The magnification are shown in Figure 8, which is a WDS elements distribution map.

The presence of these precipitates indicates the solubility of a small amount of bismuth in solid phase of zinc. Closer analysis of the Bi–Zn phase equilibrium system indicates such a possibility and additionally, the examined crystallization process took place under conditions far from the state of equilibrium. After crystallization, while the alloy was cooling, the secondary precipitation occurred, but since it occurred in the solid phase, the centrifugal force was not able to displace the precipitated particles, hence their even distribution in this part of the sample.

### 3.2. Crystallographic and Texture Analysis 

**Figure 9:**
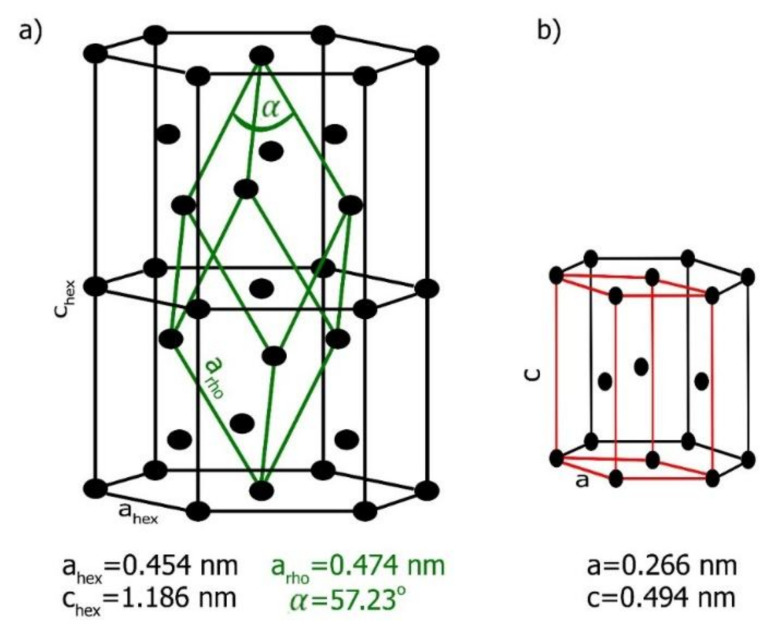
(**a**) Crystal structure of bismuth at 20 °C (rhombohedral (green) unit cell is marked into the black hexagonal lattice) and (**b**) zinc hexagonal unit cell at 20 °C (the elemental cell is marked by the red line) [30].

The crystallographic structures of zinc and bismuth are shown in Figure 9. Bismuth crystallizes in the rhombohedral structure with $R\bar{3}m$ (166) space group. The rhombohedral lattice parameter α_rho_ and the rhombohedral angle α are shown in Figure 9a.

For the undistorted reference lattice, parameters used from PDF-2 were: α_rho_ = 0.475 nm and α = 57.230°. Equivalently, the structure can be described as hexagonal with in-plane and out-of-plane lattice constants α_hex_ = 0.454 nm and c_hex_ = 1.1862 nm, where c/a = 2.6093. These lattice parameters were measured at 20 °C. Zinc crystallizes in hexagonal close pack structure with P63/mmc (194) space group. 

Additionally, there was a significant difference in lattice constants. According to Hume-Rothery rules, this excludes the possibility of the formation of solid solutions, as reflected in the appearance of the Bi–Zn phase equilibrium diagram, where solid solubility practically did not exist.

**Figure 10:**
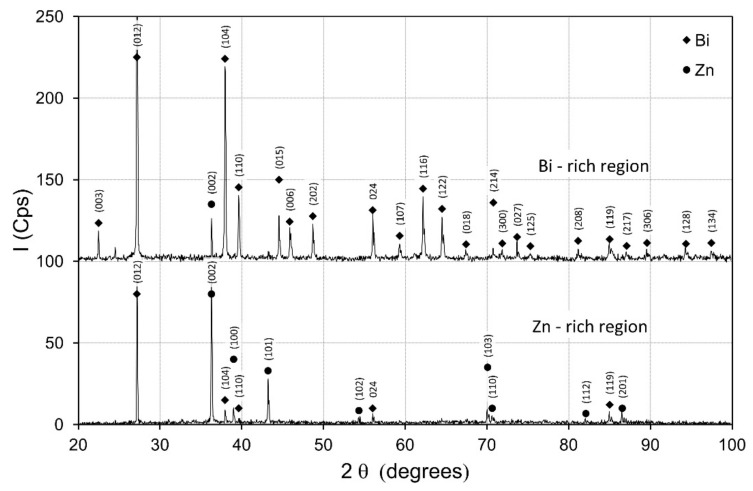
X-ray diffractograms of the Bi- and Zn-rich regions under 600 *g.*

For a thorough characteristics of the received structure, the XRD diffraction of regions rich in zinc and bismuth was carried out. The results are presented in Figure 10. In both regions of the studied specimen, only peaks characteristic for zinc and bismuth were registered. The high, dominating intensities of (002) and (101) peaks for zinc and (012) and (014) for bismuth indicate high texture of the material and appearance of dominating crystallographic orientations in both zones. The level of background in both measurements (for both parts of the sample) was homogeneous and there were no premises to discuss formation of an amorphous phase.

Regardless of the homogeneity of phases and lack of complex phases in the studied regions, their microstructure is not uniform.

**Figure 11:**
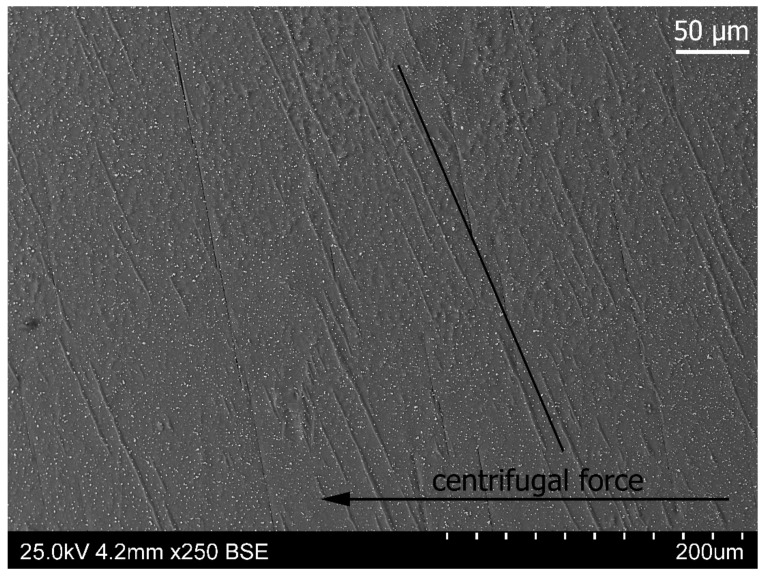
The area of needle-shaped structures in the Zn-rich zone of the sample, probably accommodation twins occurring in the central part of the sample (zones B and C, Figure 5). One preferred needle orientation suggests the existence of a highly oriented structure orientation.

Regular needle-shaped structures can be observed in areas B and C of Figure 5. That region in a higher magnification is presented in Figure 11 These needles are a result of twin deformations from stress relaxation during cooling and a simultaneous decrease in centrifugal force. Zinc as a metal with the hexagonal structure has a strong tendency of mechanical twinning [31,32]. This is indirectly caused by a small number of easy slip—only three directions $\langle 11\bar{2}0\rangle$ are lying in the (0001) base plane. Twinning structures were observed only in the right part of the sample in a rich zinc area. They were not found in an area rich in bismuth. One preferred orientation observed in Figure 11 suggests the existence of a highly ordered structure orientation.

**Figure 12:**
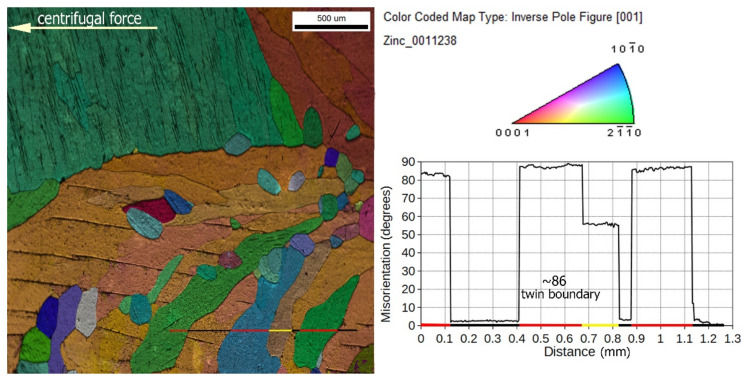
EBSD and misorientation profile obtained in the center of the 600 *g* gravity sample, marked in Figure 5 as zone C (Zn-rich zone). EBSD colored by overlapping of inverse pole figure (IPF) and band contrast (BC) images.

To confirm this concept, an EBSD analysis was performed for area C in Figure 5. The result is shown in Figure 12 in contrasting colors by overlapping of inverse pole figure (IPF) and band contrast (BC) images. A misorientation analysis (Figure 12) was carried out, which showed the existence of areas with a mutual angle of disorientation of about 86 degrees. This angle is characteristic for twinning in zinc [33] for identification of the ordered coniferous structures observed in the upper left corner of Figure 12. Their macromorphology is similar to those observed in Figure 11. Closer crystallographic analysis of these structures requires the use of high-resolution microscopy, which is planned in the next stages of research.

The studied area was characterized by a coarse-grained structure with an orientation close to ⟨0001⟩ or $\langle 10\bar{1}0\rangle$ regions, depicted in IPF representation by orange or blue, respectively (Figure 12). It is worth noting that $\langle 10\bar{1}0\rangle$ is parallel to the direction of centrifugal force.

**Figure 13:**
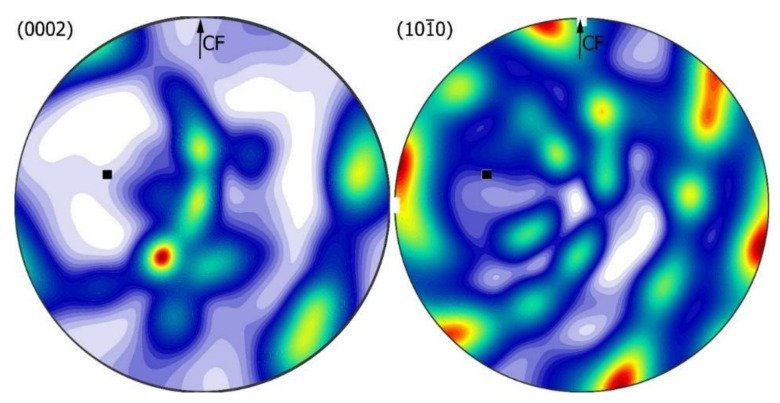
Texture analysis Zn-rich zone for 1 *g* gravity variant: full pole figures on the (0002) and $\left(10\bar{1}0\right)$ planes, recalculated in ODF. The measurement was performed with a longitudinal section of the sample.

**Figure 14:**
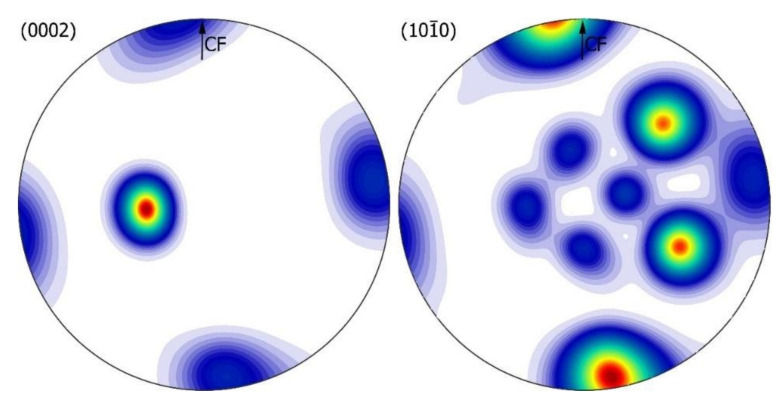
Texture analysis Zn-rich zone for 600 *g* gravity variant: full pole figures on the (0002) and $\left(10\bar{1}0\right)$ planes, recalculated in ODF. The measurement was performed with a longitudinal section of the sample.

For a more complete picture of crystallographic dependencies, the macrotexture of the Zn phase was measured by XRD. Two pole figures were measured respectively: (0002) and $\left(10\bar{1}0\right)$. Then, the orientation distribution function (ODF) was calculated in a Matlab-MTEX environment [34]. The results are presented in Figure 13 and Figure 14 in the form of full pole figures on the (0002) and $\left(10\bar{1}0\right)$ planes. 

The texture obtained for the sample at 1 *g* (normal gravity) has a character of a fibre texture $\langle 10\bar{1}0\rangle$ type, where the poles of planes (0002) and $\left(10\bar{1}0\right)$ are arranged along small circles (Figure 13). The axis of this texture (shown by the black square) is tilted approximately 76° from the CF direction.

The texture obtained for the sample at 600 *g* (supergravity variant) (Figure 14) has a strong character with two clearly outlined components with mutual twin correlation typical of hexagonal structures [33,35,36]. The main component with the ⟨0002⟩ axis tilted by ~10 degrees from CF and the growth direction parallel to $\langle 10\bar{1}0\rangle$ and a weaker component with mutual twin orientation of four peaks along the circumference on figure (0002). This observation confirms the EBSD microtexture analyses carried out for the macro area.

**Figure 15:**
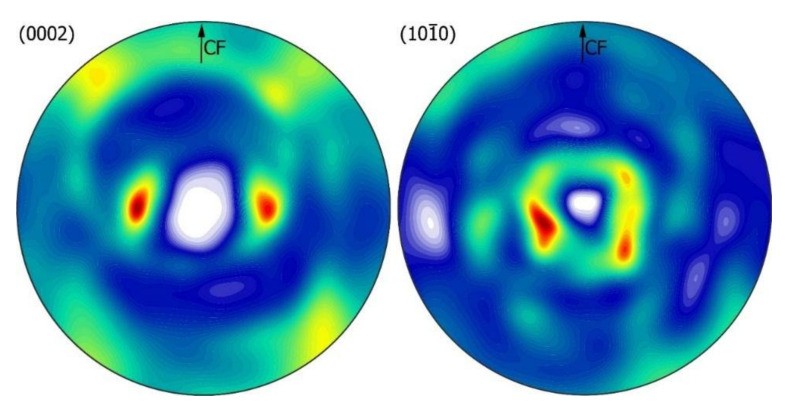
Texture analysis Bi-rich zone for 600 *g* gravity variant: full pole figures on the (0002) and $\left(10\bar{1}0\right)$planes, recalculated in ODF. The measurement was performed with a longitudinal section of the sample.

In the pole figure (0002) Bi (Figure 15), two strong peaks can be seen in a plane normal to the direction of the CF force at a distance of about 40° from the center of the pole figure. On the other hand, in the pole figure $\left(10\bar{1}0\right)$, an increase in intensity around the normal of the figure is observed in a distributed small circle with a radius of 25° (Figure 15).

The analysis of the texture of both sample zones shows that the interface was formed by two structures in a specific crystallographic relationship. The interfacial boundary has a complex character due to the strong gradient of the chemical composition in its area. This resulted in the occurrence of side-by-side microstructures belonging to opposite ends of the Zn–Bi equilibrium system.

## 4. Conclusions

The obtained results show the existence of the influence of gravity on the directed growth of the structure in Zn–Bi alloys. Analogous results obtained for other hexagonal alloys as Zn–Ti–Cu [13,14] subjected to the Bridgman zone crystallization method indicate a similar influence of the force of gravity and the direction of heat dissipation on the structure growth process. In the case of crystallization in supergravity conditions, a structure with two dominant orientations was obtained, the disorientation angle of which corresponds to the geometry of the twinning process in zinc. This effect was obtained under volumetric cooling conditions, without the forced temperature gradient as it was in the Bridgman process. The combination of zone crystallization and the force of gravity can give an interesting effect of organizing the structure. This process can be used in the production of complex materials using massive joints with controlled geometry.

Generally, studies of crystallization under 600 *g* supergravity conditions carried out on a monotectic Zn-25 wt% Bi alloy have shown the following:

- Gravity force strongly affects the nucleation and growth kinetics of eutectics;
- The boundary between the zinc-rich (Zn) and bismuth-rich (Bi) zones obtained for crystallization under supergravity is flat, while in the case of equilibrium crystallization, it is paraboloidal according to the curvature of the crystallization front;
- Crystallization under supergravity conditions results in high crystallographic order of the structure at both micro- and macro-scale;
- The (Zn)/(Bi) interface is formed by two structures in a specific crystallographic relationship;
- The strong gradient of the chemical composition near the separation boundary was observed;
- The microhardness of the zinc-rich area was about 58.5 HV and in the bismuth-rich area, it was decreased to 17.6 HV;
- The zinc-rich zone contained minor precipitates of parent bismuth, resulting from the mutual Bi–Zn solubility in a non-equilibrium state;
- The twins relation dominates in (Zn) crystallized under supergravity.

## Data Availability

The data presented in this study are available on request from the corresponding author.
